# Decoding clinical heterogeneity and age-related coronary risk in incomplete Kawasaki disease: a multicenter cluster analysis and structural equation modeling study

**DOI:** 10.3389/fimmu.2026.1908370

**Published:** 2026-09-18

**Authors:** Chang Liu, Dong Liu, Bin Li, Yanxia Xu, Zhongyou Tan, Ouyang Chen, Yanfeng Yang, Feifei Si, Wenliang Jiang, Qijian Yi, Fengchuan Jing

**Affiliations:** 1 Department of Cardiovascular Medicine, Children’s Hospital of Chongqing Medical University, Chongqing, China; 2 National Clinical Research Center for Children and Adolescents’ Health and Diseases, Children’s Hospital of Chongqing Medical University, Chongqing, China; 3 Ministry of Education Key Laboratory of Child Development and Disorders, Children’s Hospital of Chongqing Medical University, Chongqing, China; 4 Chongqing Municipal Health Commission Key Laboratory of Children’s Vital Organ Development and Diseases, Children’s Hospital of Chongqing Medical University, Chongqing, China; 5 National Clinical Key Cardiovascular Specialty, Children’s Hospital of Chongqing Medical University, Chongqing, China; 6 Department of Pediatrics, The Affiliated Hospital of Southwest Medical University, Sichuan Clinical Research Center for Birth Defects, Luzhou, China; 7 Department of Cardiovascular Medicine, Kunming Children’s Hospital, Yunnan, China; 8 Department of Pediatric Cardiovascular Medicine, Guiyang Maternal and Child Health Care Hospital, Guiyang Children’s Hospital, Guizhou, China; 9 Department of Pediatric Cardiovascular Medicine, Chongqing University Three Gorges Hospital, Chongqing, China; 10 Department of Pediatric Cardiovascular Medicine, Chengdu Women’s and Children’s Central Hospital, School of Medicine, University of Electronic Science and Technology of China, Sichuan, China; 11 Department of Pediatric Cardiovascular Medicine, Dazu Hospital of Chongqing Medical University, Chongqing, China

**Keywords:** cluster analysis, coronary artery aneurysm, incomplete Kawasaki disease, Kawasaki disease, pretreatment fever duration, subphenotype

## Abstract

**Objectives:**

To identify pretreatment clinical subphenotypes in incomplete Kawasaki disease (iKD) and examine their associations with post-IVIG coronary outcomes.

**Methods:**

We retrospectively analyzed 1,619 iKD patients from six centers. Hierarchical clustering was performed using 11 pretreatment clinical and laboratory variables, with coronary measures excluded from cluster derivation. Post-IVIG coronary outcomes were subsequently compared across clusters. Logistic regression, exploratory structural equation modeling (SEM), and LOESS analyses were used to examine factors associated with coronary artery aneurysm (CAA). The same clustering framework was also applied to the entire KD cohort (n=6,264). Focused sensitivity analyses excluded patients whose retrospective iKD classification depended on coronary findings and separately evaluated pre-IVIG coronary abnormalities and newly developed or worsening abnormalities after IVIG.

**Results:**

Four clinically distinct iKD clusters were identified. C1 showed greater systemic inflammation, C2 was characterized by younger age, longer pretreatment fever duration, and marked thrombocytosis, C3 by prominent hepatobiliary involvement, and C4 by a relatively low-inflammatory profile. C2 had the highest CAA prevalence (68.9%) and greatest coronary burden, whereas C3 had the highest IVIG-resistance rate (12.7%). Younger age, longer pretreatment fever duration, fewer clinical manifestations, lower albumin, lower hemoglobin Z-score, and lower neutrophil ratio remained independently associated with CAA. SEM showed a strong direct association of age with CAA and only a small indirect association through symptom count and pretreatment fever duration. In the whole-KD analysis, iKD was unevenly distributed across the independently derived clusters. The major cluster characteristics and the greater post-IVIG coronary burden of C2 remained evident after exclusion of patients whose iKD classification depended on coronary findings.

**Conclusions:**

iKD comprises clinically heterogeneous pretreatment phenotypes associated with different coronary and treatment-response profiles. The coronary vulnerability of younger patients appears multifactorial rather than explained solely by longer pretreatment fever duration.

## 1 Introduction

As a primary determinant of pediatric cardiac health, Kawasaki disease (KD) is an acute inflammatory disorder of the medium-sized vessels that occurs most frequently in infants and young children (1–3). While timely therapeutic management with intravenous immunoglobulin (IVIG) and aspirin has transformed the prognosis, reducing the prevalence of coronary artery aneurysms (CAAs) from 25% to nearly 5%, the disease still poses a risk of sudden cardiac complications (1, 4–6). The fundamental challenge persists in the diagnostic process, which remains entirely dependent on recognizing a constellation of physical signs. This reliance on subjective criteria often results in missed windows for intervention, especially in cases where the full diagnostic criteria are not met (1, 7). Recent evidence has further associated younger age and delayed IVIG administration with more severe coronary involvement (8).

Historically, KD has been treated as a single, homogenous disease entity. However, emerging evidence highlights profound clinical heterogeneity, prompting a shift toward unsupervised, data-driven subphenotyping to achieve precision medicine. Wang et al. pioneered this approach by identifying four distinct clinical subgroups in a US cohort, revealing that these varied clinical presentations are closely associated with distinct proteomic signatures (9). Subsequent studies have sought to validate and expand upon these findings across different populations. Gong et al. confirmed the reproducibility of this clustering model in a Chinese cohort, corroborating the existence of specific hepatic and age-related phenotypes (10). Moreover, research has increasingly focused on high-risk and temporal dynamics. Sunaga et al. conducted a targeted cluster analysis on Japanese KD patients who developed CAA, revealing that even within this severely affected subset, the clinical backgrounds and treatment responses remain highly heterogeneous (11). Adding another critical dimension, Fujiwara et al. analyzed spatiotemporal trends in Japan, showing that distinct clinical clusters exhibited unique seasonal patterns (12).

While these milestone studies have successfully mapped the broader phenotypic landscape of KD, the specific heterogeneity within the incomplete KD (iKD) population remains underexplored. It remains unclear whether clinically distinct subphenotypes exist within iKD, whether these subphenotypes are associated with different coronary outcomes and IVIG responsiveness, and which clinical characteristics distinguish patients at particularly high coronary risk. The reliance on classic clinical signs for diagnosis poses a significant challenge for iKD populations, particularly young infants, who frequently lack these classic symptoms. Consequently, these patients may experience longer intervals from fever onset to treatment, and a disproportionately high risk of CAA (1, 13, 14). Given their atypical presentations and extreme vulnerability, examining iKD separately may help characterize heterogeneity within this clinically challenging population.

To address these questions, we performed an unsupervised cluster analysis in a large multicenter cohort of patients with iKD. Our primary objective was to identify clinically distinct subphenotypes using pretreatment clinical and laboratory variables and to compare their subsequent coronary outcomes and IVIG responsiveness. We further examined pretreatment factors associated with CAA and used exploratory SEM and LOESS analyses to characterize the relationships among age, clinical manifestation burden, pretreatment fever duration, and illness-duration-related biomarker patterns. Finally, the same clustering framework was independently applied to the entire KD cohort to evaluate how iKD was distributed within the broader KD phenotypic landscape.

## 2 Methods

### 2.1 Study design and participants

This retrospective multicenter cohort study was conducted across six pediatric tertiary centers in Southwest China: Children’s Hospital of Chongqing Medical University, Kunming Children’s Hospital, Guiyang Maternal and Child Health Care Hospital, Chongqing University Three Gorges Hospital, Chengdu Women’s and Children’s Central Hospital, and Dazu Hospital of Chongqing Medical University. The study protocol was approved by the Ethics Committee of the Children’s Hospital of Chongqing Medical University and the institutional review boards of all participating sites. From an initial pool of 6,264 patients diagnosed with KD between 2015 and 2019, 1,619 patients meeting the criteria for iKD constituted the primary analytic cohort. The entire cohort of 6,264 patients with KD, including both complete KD (cKD) and iKD, was additionally retained for a secondary whole-cohort clustering analysis to place the iKD-derived phenotypes within the broader phenotypic context of KD.

Detailed eligibility criteria, including comprehensive inclusion and exclusion parameters, are provided in the **Supplementary Material** (**Supplementary Text S1**).

To ensure diagnostic consistency, the source cohort of patients clinically diagnosed with KD between 2015 and 2019 was retrospectively re-evaluated using the 2020 Japanese Diagnostic Guidelines (6th revised edition) (15).The Japanese criteria were applied as a standardized research classification framework to distinguish complete from incomplete clinical presentations. The six principal clinical features considered were fever, bilateral conjunctival injection, changes of the lips or oral cavity, rash, changes of the peripheral extremities, and nonsuppurative cervical lymphadenopathy. A principal clinical feature was considered present if it had been documented at any time before initiation of the first IVIG infusion. Fever was counted as one principal feature, whereas the symptom-count variable represented the number of the remaining five non-fever manifestations.

According to the 2020 Japanese criteria, patients with three principal features accompanied by a coronary artery Z-score ≥2.5 were classified as iKD, whereas patients with three to four principal features without coronary dilation could be classified as iKD when other significant supportive findings were present after exclusion of alternative febrile illnesses. Patients with highly incomplete presentations (≤2 principal clinical features) were reviewed using the available pretreatment clinical record because the Japanese guideline does not provide a fixed numerical diagnostic threshold for this subgroup. For reconstruction of the pretreatment diagnostic pathway, all echocardiographic examinations performed before the first IVIG infusion were reviewed, and the maximum coronary Z-score observed during this period was used to determine pretreatment coronary status. Pretreatment coronary findings used for retrospective classification were analyzed separately from post-IVIG coronary measurements used as study outcomes.

Raw coronary dimensions were transformed into standardized Z-scores using the Kobayashi et al. regression equations (16). Echocardiographic examinations were performed as part of routine clinical care at each participating center. Because this was a retrospective multicenter study, the exact timing and frequency of echocardiographic examinations were not prospectively standardized and were determined by the treating physicians according to each patient’s clinical condition and coronary findings. For the present analysis, only echocardiographic measurements obtained after completion of the initial IVIG infusion and before hospital discharge were used for coronary outcome assessment. The maximum post-IVIG Z-score observed in any major coronary artery branch between completion of initial IVIG and hospital discharge was used as the coronary Z-score outcome. Coronary artery status was stratified into five distinct severity tiers, the definitions and Z-score thresholds of which are detailed in the **Supplementary Material** (**Supplementary Text S1**). IVIG resistance was defined as persistent fever with a body temperature ≥37.5 °C at or beyond 36 hours after completion of the initial IVIG infusion, or recrudescent fever ≥37.5 °C occurring after an initial period of defervescence at or beyond 36 hours after IVIG completion. Classification was based on the febrile response to initial IVIG and was independent of subsequent rescue therapy.Fever duration was defined as the interval, in days, from the onset of fever to initiation of the first IVIG infusion.

### 2.2 Data collection and preprocessing

Baseline clinical and laboratory data before initial IVIG treatment were extracted from electronic medical records. Eleven continuous variables were selected for cluster analysis based on clinical relevance and prior literature (9–12): age at onset, fever duration before treatment (days), white blood cell count (WBC), platelet count (PLT), C-reactive protein (CRP), serum albumin (ALB), alanine aminotransferase (ALT), gamma-glutamyl transferase (GGT), hemoglobin Z-score (HB), neutrophil ratio (NR), and erythrocyte sedimentation rate (ESR). The selection was intended to provide a parsimonious representation of complementary pretreatment clinical domains, including age, illness duration, systemic inflammation, hematologic response, and hepatobiliary involvement, while maintaining a homogeneous set of quantitative variables for clustering. This strategy was broadly consistent with previous data-driven KD clustering studies, in which clustering was primarily based on continuous demographic and laboratory variables, while categorical clinical manifestations were evaluated separately after cluster formation (9–11). Individual KD manifestations and total symptom count were therefore not included as clustering variables. Because these manifestations constitute components of the diagnostic definition of complete versus incomplete KD, particularly within an iKD-only cohort, including them in cluster derivation could have caused the resulting partition to reflect the number or pattern of diagnostic criteria rather than underlying quantitative clinical heterogeneity. Instead, individual manifestations and total symptom count were retained as independent clinical descriptors and were compared across the derived clusters.

To ensure the independence of the selected features, pairwise collinearity was assessed using Spearman correlation analysis. All correlation coefficients (|r|) between the 11 continuous variables were below 0.70, indicating that no significant multicollinearity existed and that each variable provided distinct information for the clustering algorithm.

To handle missing values (missing rate < 20%), we employed the missForest algorithm, a robust non-parametric random forest-based imputation method, which provides superior accuracy for clinical datasets (17). Subsequently, all continuous variables were standardized using Z-score normalization (scaling) to ensure equal weight in the clustering process.

### 2.3 Clustering analysis pipeline

We performed an unsupervised, data-driven hierarchical cluster analysis to identify clinical clusters. The clustering tendency was first assessed using the Hopkins statistic via the factoextra package (18, 19), with a value exceeding 0.5 indicating a tendency toward a non-random clustering structure. The optimal number of clusters was determined through a multi-index approach, integrating the elbow method and the NbClust package (20, 21), which evaluates 30 distinct indices. Hierarchical clustering was then conducted using Euclidean distance with Ward’s D2 linkage method to minimize total within-cluster variance (22, 23). The resulting dendrogram was then partitioned into four clusters according to the optimal cluster number determined by the preceding multi-index evaluation. Finally, principal component analysis (PCA) was performed using the FactoMineR package to visualize the cluster distribution in a two-dimensional space and to identify the primary clinical drivers contributing to heterogeneity (24).

To examine whether the identified patterns were specific to iKD, the same 11-variable pretreatment clustering framework was independently applied to the entire KD cohort (n=6,264), including both cKD and iKD. The same imputation, standardization, cluster-number evaluation, Euclidean-distance/Ward’s D2 hierarchical clustering, and visualization procedures were used, and the optimal cluster number was redetermined within the whole KD cohort rather than imposed from the iKD analysis. After cluster derivation, the proportion of iKD patients and subsequent coronary outcomes, including CAA prevalence, maximum post-IVIG coronary Z-score, and coronary severity, were compared across the whole-cohort clusters. This analysis was used to determine whether iKD was disproportionately represented within particular regions of the broader KD clinical spectrum and whether the whole-cohort clusters were associated with independently assessed coronary outcomes.

### 2.4 Statistical analysis

Continuous variables were assessed for normality using the Kolmogorov-Smirnov test. As the continuous variables showed non-normal distributions, they were presented as median (interquartile range [IQR]) and analyzed using non-parametric methods. The Kruskal-Wallis H test was employed for global comparisons across the four clusters, followed by Dunn’s *post-hoc* test with Benjamini-Hochberg correction for pairwise inter-group comparisons. Categorical variables were presented as n (%) and were compared using the Chi-squared or Fisher’s exact test. Violin plots were generated using GraphPad Prism (version 8.2.1; GraphPad Inc., CA, USA). Stacked and faceted bar charts illustrated clinical outcomes.

To evaluate pretreatment factors associated with post-IVIG CAA, univariable logistic regression analyses were performed with CAA as the dependent variable, and odds ratios (ORs) with 95% confidence intervals (CIs) were reported. Total symptom count was used as the summary measure of clinical manifestations in the regression analyses, while individual manifestations were evaluated descriptively. Variables associated with CAA at P < 0.05 in univariable analyses were entered simultaneously into the multivariable model using a forced-entry approach.

Given their clinically plausible temporal relationship, age, total symptom count, and pretreatment fever duration were further examined in an exploratory SEM to characterize their direct and indirect associations with CAA. SEM was performed using the lavaan R package with the weighted least squares mean- and variance-adjusted (WLSMV) estimator, with CAA modeled as an ordered binary outcome (25, 26) Model fit was assessed using the comparative fit index (CFI), Tucker–Lewis index (TLI), standardized root mean square residual (SRMR), and root mean square error of approximation (RMSEA), together with the scaled chi-square statistic. Given the observational study design, path directions were specified on clinical grounds and all direct and indirect effects were interpreted as exploratory associations rather than evidence of causality or formal causal mediation.

Furthermore, locally estimated scatterplot smoothing (LOESS) regression analyzed the cross-sectional patterns of laboratory markers relative to fever duration. Specifically, CRP and PLT were examined against pretreatment fever duration in the pooled iKD cohort, with the analysis restricted to illness days 1–15 to avoid undue influence from sparsely represented extreme illness durations. LOESS-fitted curves with 95% confidence intervals were used to visualize the cross-sectional relationships. This analysis was intended to assess whether prominent CRP–PLT differences contributing to cluster characterization might partly reflect illness-duration-related variation; it was not used to infer within-patient longitudinal progression or transition between clusters. Except for violin plots, all analyses were performed in R (v4.3.2). A two-tailed P-value < 0.05 was considered statistically significant.

Focused sensitivity analyses were performed to address potential incorporation and selection bias related to retrospective iKD classification. First, patients whose iKD classification explicitly depended on coronary findings were excluded, leaving a restricted coronary-independent cohort of 1,311 patients. Clustering tendency was reassessed using the Hopkins statistic, and the same 11 pretreatment variables, standardization procedure, Euclidean distance, and Ward’s D2 hierarchical clustering method were reapplied using the prespecified four-cluster framework from the primary analysis to facilitate direct comparison of phenotypic patterns. Coronary outcomes were subsequently compared across the resulting clusters. Second, coronary abnormalities present before initial IVIG were distinguished from abnormalities that newly developed or worsened during the post-IVIG predischarge period. Newly developed CAA was defined as a post-IVIG coronary Z-score ≥2.5 among patients with a pre-IVIG Z-score <2.5. Worsening of pre-existing CAA was conservatively defined as progression to a higher predefined coronary severity category after IVIG. In addition, the change in maximum coronary Z-score (ΔZ), calculated as the maximum post-IVIG Z-score minus the maximum pre-IVIG Z-score, was examined as a continuous measure among patients with pre-IVIG CAA.

Because this was a retrospective multicenter study including all eligible patients during the predefined study period, no *a priori* sample-size calculation was performed. A *post-hoc* power analysis was subsequently conducted for the comparison of CAA prevalence across the four identified clusters. Based on the observed effect size (Cohen’s w = 0.138), a total sample size of 1,619, three degrees of freedom, and an α level of 0.05, the achieved statistical power exceeded 99.9%.

## 3 Results

### 3.1 Baseline characteristics of the study population

From an initial screening of 6,264 patients diagnosed with KD, a total of 1,619 iKD patients were enrolled in the final analysis after applying rigorous inclusion and exclusion criteria.

Retrospective reconstruction of the pretreatment diagnostic pathway identified 308 patients (19.0%) whose iKD classification explicitly required coronary findings, defined as three principal clinical features accompanied by a pre-IVIG coronary artery Z-score ≥2.5. The remaining 1,311 patients (81.0%) were classified as iKD without requiring coronary findings and constituted the coronary-independent cohort used for the focused sensitivity analysis.

The median age was 1.2 years (IQR, 0.6–1.9 years), and 1,015 (62.7%) patients were male. The median pretreatment fever duration was 7 days (IQR, 6–9 days). Overall, 873 (53.9%) patients had post-IVIG CAA, and 133 (8.2%) exhibited IVIG resistance.

### 3.2 Identification of four clinical clusters in iKD

**Figure 1:**
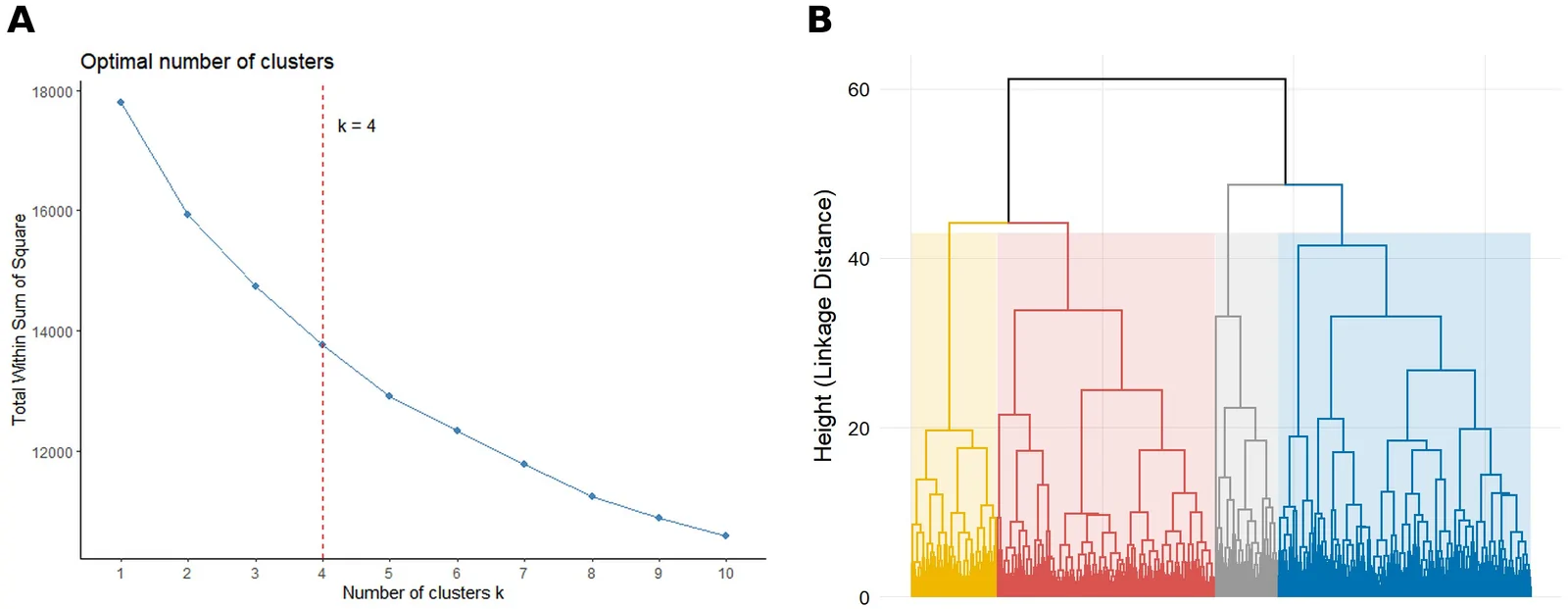
Determination of the optimal number of clusters and hierarchical clustering of the iKD cohort. **(A)** Elbow plot showing the total within-cluster sum of squares across candidate cluster numbers. The red dashed line indicates the selected four-cluster solution (k=4), supported by the multi-index cluster-number evaluation. **(B)** Hierarchical clustering dendrogram of 1,619 patients with incomplete Kawasaki disease (iKD), constructed using the 11 pretreatment clinical and laboratory variables with Euclidean distance and Ward’s D2 linkage. The four derived clusters are indicated by distinct branch and background colors. Abbreviation: iKD, incomplete Kawasaki disease.

The Hopkins statistic was 0.856, indicating a clustering tendency within the iKD cohort. Evaluation of the cluster number using the elbow method and the multi-index approach implemented in NbClust supported selection of a four-cluster solution (**Figure 1**). Hierarchical clustering based exclusively on the 11 pretreatment clinical and laboratory variables classified 659 patients into Cluster 1 (C1), 225 into Cluster 2 (C2), 165 into Cluster 3 (C3), and 570 into Cluster 4 (C4).

**Figure 2:**
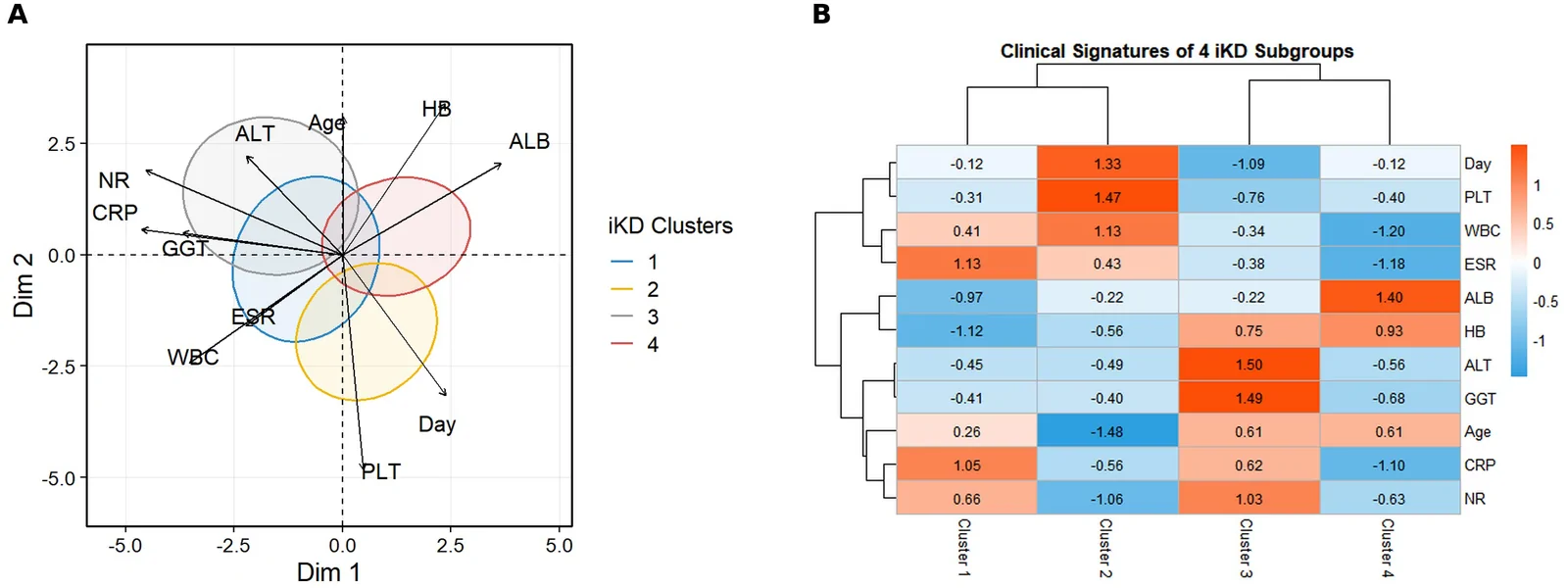
Principal component and heatmap visualization of clinical heterogeneity across the four iKD Clusters. **(A)** Principal component analysis (PCA) biplot illustrating the distribution of the four clusters in the first two principal-component dimensions. Shaded ellipses represent the cluster distributions, and arrows indicate the direction and magnitude of loadings of the 11 pretreatment variables. **(B)** Standardized heatmap showing the clinical and laboratory profiles of the four clusters. Cell values represent standardized cluster-level values, with red indicating relatively higher and blue indicating relatively lower levels. Abbreviations: iKD, incomplete Kawasaki disease; PCA, principal component analysis; WBC, white blood cell count; PLT, platelet count; CRP, C-reactive protein; ALB, albumin; ALT, alanine aminotransferase; GGT, gamma-glutamyl transferase; HB, hemoglobin Z-score; NR, neutrophil ratio; ESR, erythrocyte sedimentation rate; Day, pretreatment fever duration.

PCA demonstrated partially overlapping but different distributions of the four clusters, whereas the standardized heatmap showed distinct pretreatment clinical and laboratory patterns across clusters (**Figure 2**). The principal features contributing to cluster characterization included inflammatory markers, hepatic enzymes, platelet count, age, and pretreatment fever duration. Importantly, no coronary variable was included in cluster derivation.

### 3.3 Clinical characteristics and coronary outcomes across the four iKD clusters

**Figure 3:**
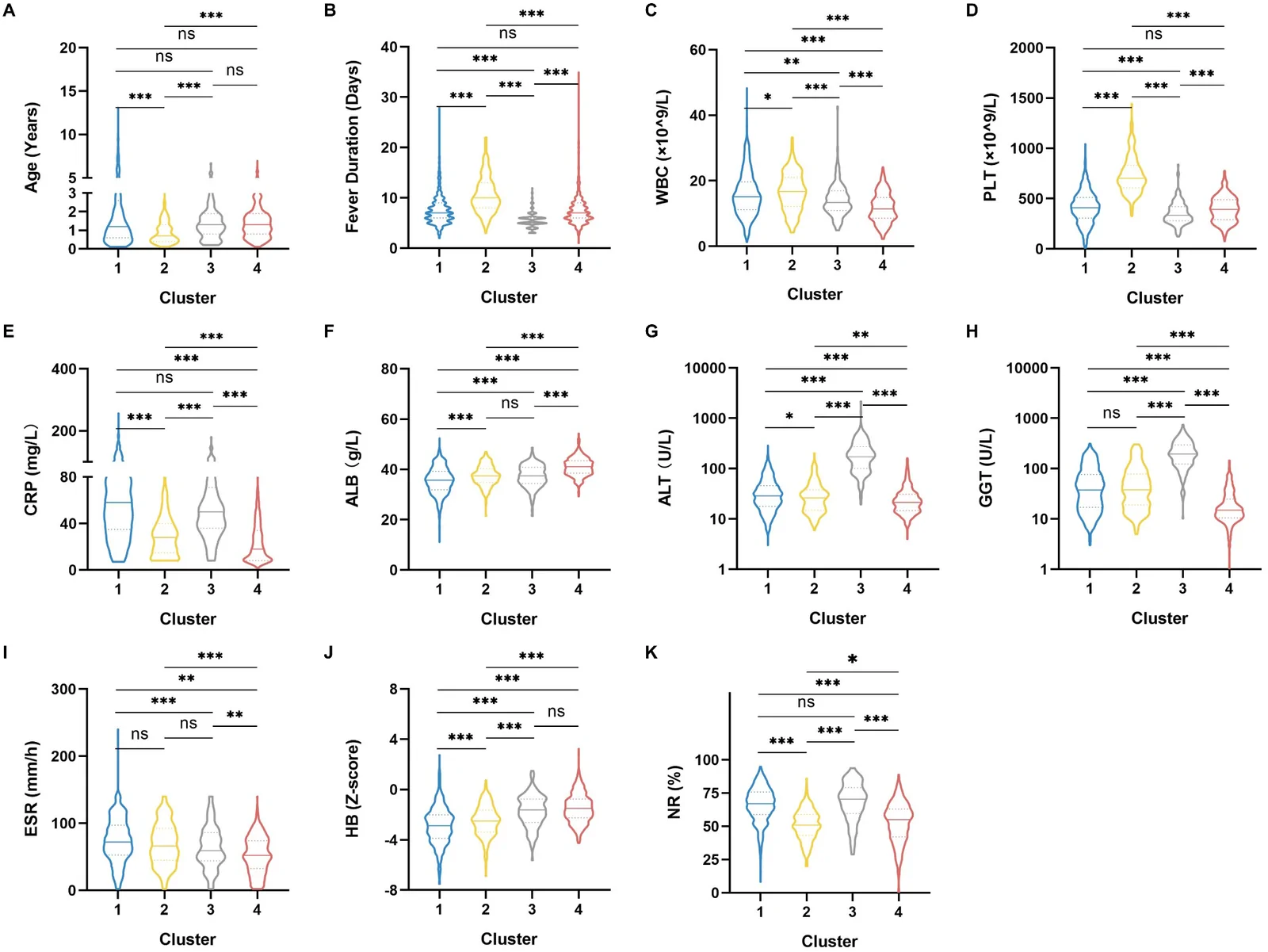
Distribution of pretreatment clinical and laboratory variables across the four iKD clusters. Violin plots with internal median and interquartile-range lines show the distributions of the 11 continuous variables used for cluster characterization: **(A)** age, **(B)** pretreatment fever duration, **(C)** WBC, **(D)** PLT, **(E)** CRP, **(F)** ALB, **(G)** ALT, **(H)** GGT, **(I)** ESR, **(J)** hemoglobin Z-score, and **(K)** NR. Pairwise comparison annotations are based on *post-hoc* comparisons following the overall between-cluster test. *P<0.05; **P<0.01; ***P<0.001; ns, not significant. iKD, incomplete Kawasaki disease; WBC, white blood cell count; PLT, platelet count; CRP, C-reactive protein; ALB, albumin; ALT, alanine aminotransferase; GGT, gamma-glutamyl transferase; ESR, erythrocyte sedimentation rate; NR, neutrophil ratio.

**Figure 4:**
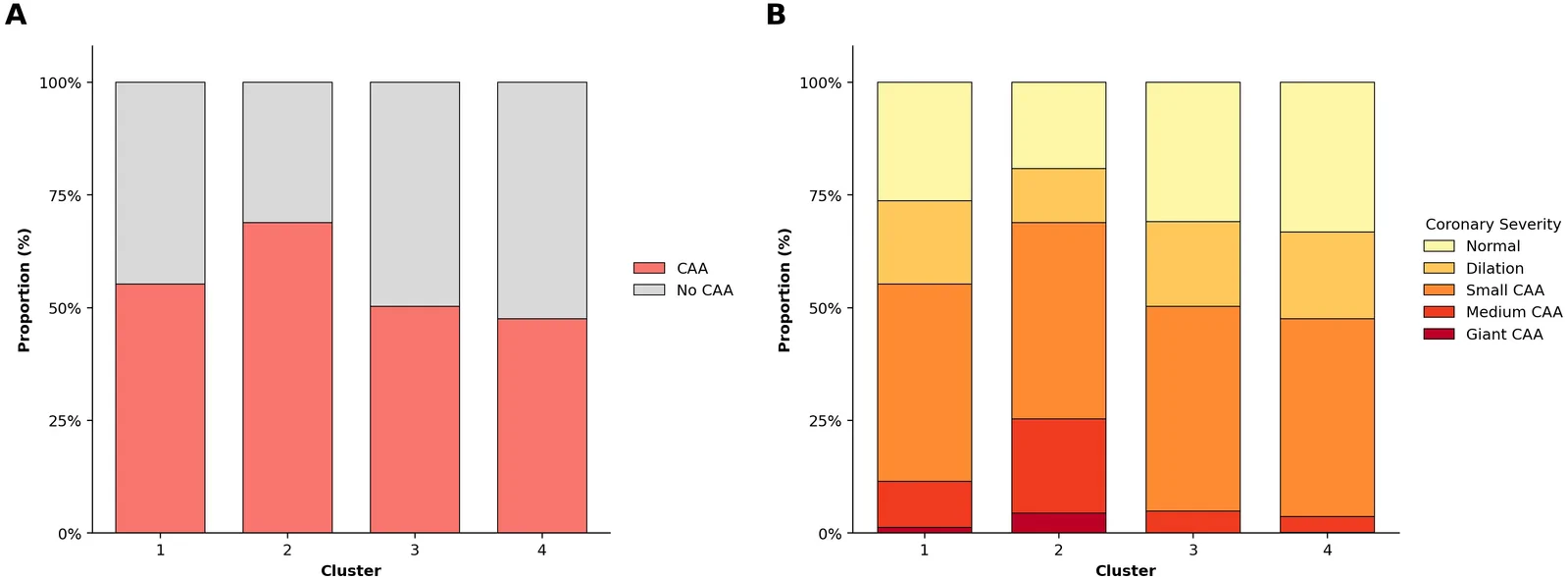
Post-IVIG coronary outcomes across the four iKD clusters. Stacked bar charts show **(A)** the proportion of patients with and without coronary artery aneurysm (CAA) and **(B)** the distribution of coronary severity categories across the four clusters. Coronary status was determined using the maximum coronary artery Z-score recorded after completion of the initial IVIG infusion and before hospital discharge. Severity categories were classified as normal, dilation, small CAA, medium CAA, and giant CAA according to the predefined Z-score criteria. Between-cluster differences in both CAA prevalence and coronary severity distribution were significant (P<0.001). Abbreviations: iKD, incomplete Kawasaki disease; CAA, coronary artery aneurysm; IVIG, intravenous immunoglobulin.

**Figure 5:**
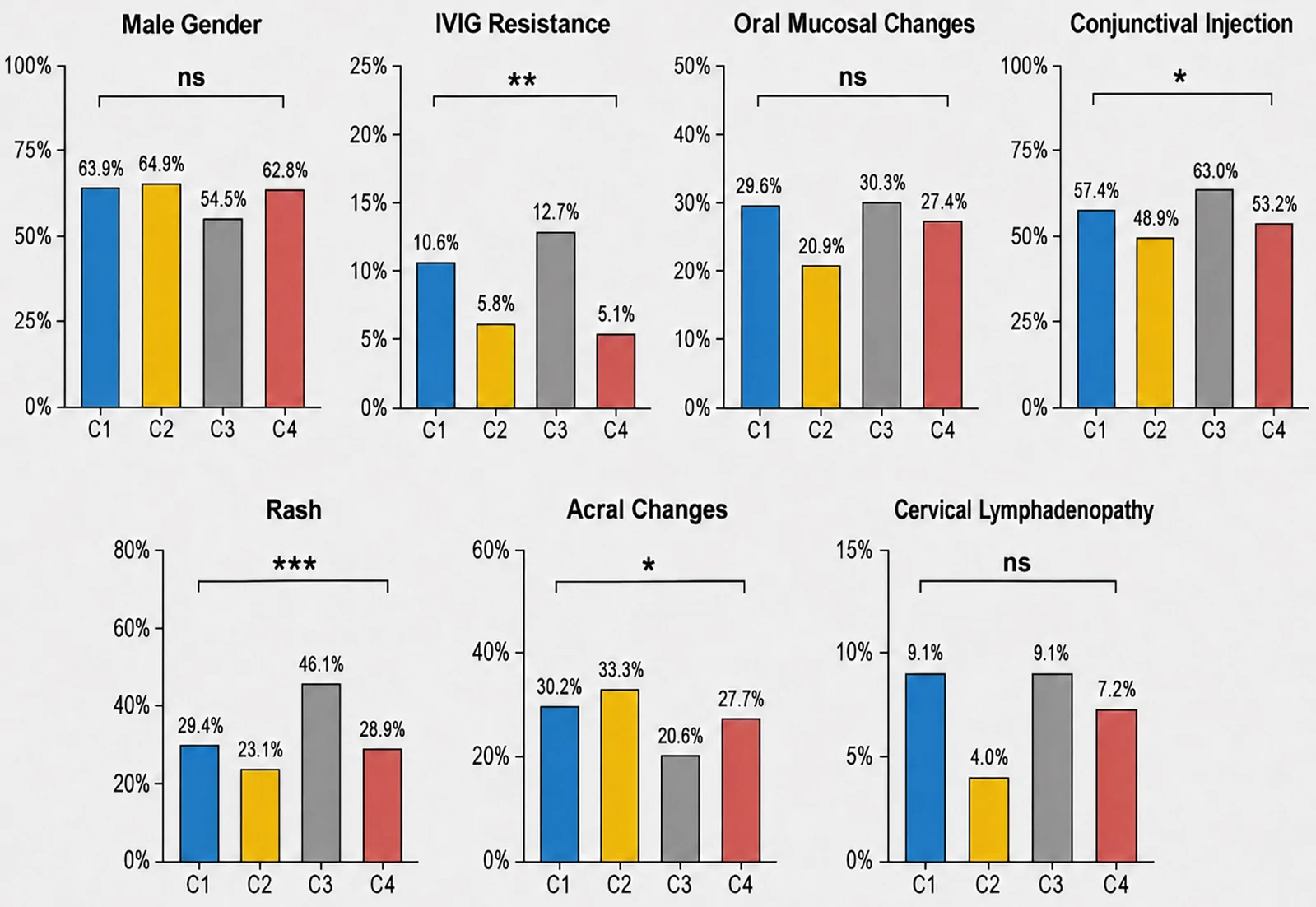
Clinical manifestations and IVIG resistance across the four iKD clusters. Bar charts show the proportions of male patients, IVIG resistance, and the five principal clinical manifestations of Kawasaki disease across the four clusters. Symbols above each panel indicate the overall between-cluster comparison using the Chi-squared test or Fisher’s exact test, as appropriate. *P<0.05; **P<0.01; ***P<0.001; ns, not significant. Abbreviations: iKD, incomplete Kawasaki disease; IVIG, intravenous immunoglobulin.

The four clusters differed significantly in their pretreatment clinical and laboratory profiles (**Tables 1**, **2**; **Figures 3**–**5**). C1 was characterized by greater systemic inflammatory activity, with relatively higher CRP, NR, and ESR and lower ALB and hemoglobin Z-scores. C2 comprised the youngest patients and was characterized by the longest pretreatment fever duration, marked thrombocytosis, relatively lower CRP and NR levels, and fewer clinical manifestations. C3 was distinguished by prominent hepatobiliary involvement, with markedly elevated ALT and GGT levels and the shortest pretreatment fever duration. C4 showed a comparatively low-inflammatory profile, with lower WBC, CRP, and ESR and higher ALB.

**Table 1:** Clinical characteristics, laboratory profiles, treatment response, and coronary outcomes across the four iKD clusters.

Variable	Overall (n=1619)	1 (n=659)	2 (n=225)	3 (n=165)	4 (n=570)	P-value
Age (Years, median [IQR])	1.2 [0.6,1.9]	1.2 [0.6,2.0]	0.7 [0.4,1.3]	1.3 [0.8,1.9]	1.3 [0.8,1.9]	<0.001***
Fever Duration (Days, median [IQR])	7 [6,9]	7 [6,9]	10 [8,13]	5 [5,6]	7 [6,9]	<0.001***
WBC (10^9/L, median [IQR])	13.82 [10.12,17.90]	15.1 [11.2,19.63]	16.74 [12.22,20.96]	13.39 [11.09,16.76]	11.43 [8.57,14.88]	<0.001***
PLT (10^9/L, median [IQR])	424 [314,543]	409 [307,509.5]	701.5 [606.5,830.25]	334 [279.5,418.5]	393 [291.5,488.5]	<0.001***
CRP (mg/L, median [IQR])	36 [17,60.5]	58 [35,82]	28 [15,39.75]	50 [36,70.5]	18 [8,34]	<0.001***
ALB (g/L, median [IQR])	38.2 [34.8,41.65]	35.7 [31.9,39.2]	37.4 [34.9,40.2]	37.4 [34.4,40.7]	41.1 [38.5,43.4]	<0.001***
ALT (U/L, median [IQR])	26.5 [16.6,47.5]	28.7 [17.85,45.98]	26.3 [14.9,37.6]	171.2 [101.85,270.5]	21.3 [14.75,31]	<0.001***
GGT (U/L, median [IQR])	28 [13.8,72]	37.55 [17,76.22]	38 [19,78.7]	196.2 [124,294.48]	15 [10.5,24.7]	<0.001***
Maximum post-IVIG Z-score (median [IQR])	2.51 [1.81,3.47]	2.56 [1.87,3.6]	3.28 [2.24,4.98]	2.48 [1.75,3.24]	2.34 [1.68,3.07]	<0.001***
Hemoglobin Z-score (median [IQR])	-2.25 [-3.12,-1.25]	-2.88 [-3.88,-2]	-2.5 [-3.38,-1.62]	-1.62 [-2.59,-0.78]	-1.5 [-2.25,-0.75]	<0.001***
NR (%, median [IQR])	60.99 [50,70.97]	67.02 [58.96,75.63]	50.98 [43.74,58.97]	70.5 [59.96,79]	55 [42.05,63.03]	<0.001***
ESR (mm/h, median [IQR])	63 [42,86]	72 [53,97]	66 [45,92]	59 [44,86]	52 [33,74]	<0.001***
CAA n (%)	873 (53.9)	364 (55.2)	155 (68.9)	83 (50.3)	271 (47.5)	<0.001***
Detailed severity n (%)						<0.001†***
Normal Coronary	456 (28.2)	173 (26.3)	43 (19.1)	51 (30.9)	189 (33.2)	
Coronary Dilation	290 (17.9)	122 (18.5)	27 (12)	31 (18.8)	110 (19.3)	
Small CAA	711 (43.9)	288 (43.7)	98 (43.6)	75 (45.5)	250 (43.9)	
Medium CAA	143 (8.8)	68 (10.3)	47 (20.9)	8 (4.8)	20 (3.5)	
Giant CAA	19 (1.2)	8 (1.2)	10 (4.4)	0 (0)	1 (0.2)	
Resistance n (%)	133 (8.2)	70 (10.6)	13 (5.8)	21 (12.7)	29 (5.1)	0.005**
Male Gender n (%)	1015 (62.7)	421 (63.9)	146 (64.9)	90 (54.5)	358 (62.8)	0.136
Symptom count (median [IQR])	2 [1,2]	2 [1,2]	1 [1,2]	2 [1,2]	2 [1,2]	<0.001***
Oral mucosal changes n (%)	448 (27.7)	195 (29.6)	47 (20.9)	50 (30.3)	156 (27.4)	0.073
Conjunctival injection n (%)	895 (55.3)	378 (57.4)	110 (48.9)	104 (63)	303 (53.2)	0.019*
Rash n (%)	487 (30.1)	194 (29.4)	52 (23.1)	76 (46.1)	165 (28.9)	<0.001***
Acral changes n (%)	466 (28.8)	199 (30.2)	75 (33.3)	34 (20.6)	158 (27.7)	0.035*
Cervical lymphadenopathy n (%)	125 (7.7)	60 (9.1)	9 (4)	15 (9.1)	41 (7.2)	0.079

Continuous variables with non-normal distributions are presented as median [interquartile range, IQR], and differences among groups were evaluated using the Kruskal-Wallis H test. Categorical variables are presented as frequencies (%) and were compared using the Chi-squared test or Fisher’s exact test. *P < 0.05, **P < 0.01, ***P < 0.001. †P-value was calculated for the overall distribution of detailed coronary severity grades across the four clusters. iKD, incomplete Kawasaki disease; IQR, interquartile range; WBC, white blood cell; PLT, platelet; CRP, C-reactive protein; ALB, albumin; ALT, alanine aminotransferase; GGT, gamma-glutamyl transferase; CAA, coronary artery aneurysm; NR, neutrophil ratio; ESR, erythrocyte sedimentation rate.

**Table 2:** Defining clinical and laboratory profiles and major outcomes of the four iKD subphenotypes.

Feature	Cluster 1: Hyper-inflammatory	Cluster 2: Young-age/ thrombocytosis	Cluster 3: Hepatic-involved	Cluster 4: Low-inflammatory/ mild
Primary Feature	Systemic inflammatory activation	Young age, prolonged pretreatment fever, and marked thrombocytosis	Prominent hepatic involvement	Relatively mild inflammatory profile
Age at Onset	Moderate	**Youngest**	Moderate	Moderate
Key Lab Findings	↑ CRP, ↑ NR, ↑ ESR; relatively low ALB/HB	**↑↑ PLT;** **relatively lower CRP and NR**	**↑↑ ALT, ↑↑ GGT**	↑ ALB; ↓ CRP, ↓ WBC, ↓ ESR
Fever Duration	Moderate (7 days)	Longest (10 days)	Shortest (5 days)	Moderate (7 days)
Observed Coronary Outcome	CAA 55.2%; moderate coronary burden	**Highest CAA rate (68.9%);** **highest maximum Z-score and greatest medium/giant CAA burden**	CAA 50.3%; moderate coronary burden	Lowest CAA rate (47.5%)
IVIG Resistance Rate	Relatively high (10.6%)	Low (5.8%)	**Highest observed (12.7%)**	Lowest observed (5.1%)

This table summarizes the defining characteristics of each identified iKD cluster. Bold text highlights the most prominent clinical features for each phenotype. The arrows (↑ or ↑↑) represent higher levels relative to other clusters, with ↑↑ denoting markedly elevated values. Coronary variables were not used for the revised clustering and are shown here as subsequently evaluated outcomes. The IVIG resistance rate is retained as a clinically relevant post-clustering characteristic and is not used to define the hepatic-involved phenotype. Qualitative descriptors of IVIG resistance are descriptive rankings based on the observed rates and do not imply pairwise statistical significance. iKD, incomplete Kawasaki disease; IVIG, intravenous immunoglobulin; ALT, alanine aminotransferase; GGT, gamma-glutamyl transferase; CRP, C-reactive protein; WBC, white blood cell count; PLT, platelet count; NR, neutrophil ratio; ESR, erythrocyte sedimentation rate; ALB, albumin; HB, hemoglobin Z-score; CAA, coronary artery aneurysm.

Despite the exclusion of coronary variables from cluster derivation, subsequent coronary outcomes differed significantly across clusters. C2 showed the greatest coronary burden, with the highest CAA prevalence (68.9%), maximum post-IVIG coronary Z-score [3.28 (IQR, 2.24–4.98)], and proportions of medium and giant CAA. CAA prevalence was 55.2%, 68.9%, 50.3%, and 47.5% in C1–C4, respectively (P<0.001). C3 had the highest observed IVIG-resistance rate (12.7%), whereas C4 had the lowest CAA prevalence (47.5%) and IVIG-resistance rate (5.1%).

### 3.4 Whole-cohort analysis of clinical clustering in KD

To examine whether the clinical patterns identified within iKD were specific to incomplete disease, the same pretreatment-only clustering framework was independently applied to the entire KD cohort (n=6,264). Four clusters were identified, comprising 3,287, 1,541, 851, and 585 patients, respectively (**Supplementary Table S1**; **Supplementary Figures S1**-**S3**). The proportion of iKD differed significantly across the four clusters, accounting for 23.4%, 36.3%, 20.0%, and 20.7% of C1–C4, respectively (P<0.001), indicating disproportionate representation of iKD in C2.

Coronary outcomes also differed across the whole-cohort clusters, with CAA prevalence of 55.1%, 47.1%, 46.9%, and 25.3% in C1–C4, respectively (P<0.001); maximum post-IVIG coronary Z-score and coronary severity also differed significantly across clusters. These findings showed that iKD was unevenly distributed across the independently derived whole-KD clusters and was most strongly represented in C2, providing a broader phenotypic context for the iKD findings.

### 3.5 Focused sensitivity analyses

After excluding 308 patients whose iKD classification explicitly depended on coronary findings, 1,311 patients remained in the restricted sensitivity cohort. Reapplication of the prespecified clustering framework reproduced four clinically distinct phenotypes, including hyper-inflammatory (C1), young-age/thrombocytosis (C2), hepatobiliary-involved (C3), and relatively low-inflammatory (C4) profiles (**Supplementary Table S2**, **Supplementary Figure S4**). Coronary outcome patterns remained consistent with the primary analysis, with C2 showing the greatest CAA burden after restriction of the cohort. Temporal analyses further indicated that a substantial proportion of CAA cases were already detectable before IVIG administration, with pre-IVIG CAA being most frequent in C2. Among patients without pre-IVIG CAA, newly developed CAA after IVIG was uncommon, and neither post-IVIG CAA progression nor changes in coronary Z-score differed significantly across clusters (**Supplementary Table S3**).

### 3.6 Pretreatment factors associated With CAA

Univariable logistic regression identified younger age, longer pretreatment fever duration, higher WBC and PLT, lower ALB and hemoglobin Z-score, higher GGT, lower NR, and lower symptom count as factors significantly associated with post-IVIG CAA (**Table 3**).

**Table 3:** Univariable logistic regression of pretreatment clinical and laboratory features associated with CAA.

Variable	OR	Lower 95% CI	Upper 95% CI	P-value
Age	0.72	0.67	0.77	<0.001***
Pretreatment fever duration	1.07	1.04	1.10	<0.001***
WBC	1.03	1.01	1.05	<0.001***
PLT	1.15	1.08	1.22	<0.001***
CRP	0.99	0.96	1.03	0.728
ALB	0.94	0.92	0.96	<0.001***
ALT	0.99	0.98	1.01	0.331
GGT	1.02	1.01	1.03	0.003**
Hemoglobin Z-score	0.83	0.77	0.89	<0.001***
NR	0.98	0.97	0.99	<0.001***
ESR	0.99	0.95	1.02	0.472
Symptom count	0.78	0.71	0.86	<0.001***
Gender (male)	1.06	0.87	1.30	0.546

In this model, CAA serves as the dependent variable. ORs for continuous variables represent the change in odds per 1-year increase in age, per 1-day increase in pretreatment fever duration, per 1 ×10^9/L increase in WBC, per 100 ×10^9/L increase in PLT, per 10 mg/L increase in CRP, per 1 g/L increase in ALB, per 10 U/L increase in ALT and GGT, per 1-unit increase in hemoglobin Z-score, per 1% increase in NR, per 10 mm/h increase in ESR, and per 1-feature increase in symptom count. Maximum coronary Z-score, detailed coronary severity, and IVIG resistance were not included because they represent coronary outcome measures or post-treatment characteristics. *P < 0.05, **P < 0.01, ***P < 0.001. Abbreviations: OR, odds ratio; CI, confidence interval; CAA, coronary artery aneurysm; WBC, white blood cell; PLT, platelet; CRP, C-reactive protein; ALB, albumin; ALT, alanine aminotransferase; GGT, gamma-glutamyl transferase; NR, neutrophil ratio; ESR, erythrocyte sedimentation rate.

In the multivariable model, younger age (OR, 0.82; 95% CI, 0.75–0.89; P<0.001), longer pretreatment fever duration (OR, 1.04; 95% CI, 1.00–1.08; P = 0.041), and lower symptom count (OR, 0.83; 95% CI, 0.74–0.93; P = 0.002) remained independently associated with CAA. Lower ALB, lower hemoglobin Z-score, and lower NR also remained independently associated with CAA, whereas WBC, PLT, and GGT were no longer statistically significant after adjustment (**Table 4**).

**Table 4:** Multivariable logistic regression of factors independently associated with coronary artery aneurysm.

Variable	OR	Lower 95% CI	Upper 95% CI	P-value
Age	0.82	0.75	0.89	<0.001***
Pretreatment Fever duration	1.04	1.00	1.08	0.041*
WBC	1.02	1.00	1.04	0.114
PLT	1.05	0.97	1.13	0.235
ALB	0.95	0.92	0.98	<0.001***
GGT	1.02	1.00	1.03	0.058
HB	0.90	0.82	0.99	0.023*
NR	0.98	0.97	0.99	<0.001***
Symptom count	0.83	0.74	0.93	0.002**

CAA was utilized as the dependent variable. Variables with P < 0.05 in univariable analyses were entered into the multivariable model using a forced-entry approach; symptom count was included instead of individual clinical manifestations. *P < 0.05, **P < 0.01, ***P < 0.001. OR, odds ratio; CI, confidence interval; CAA, coronary artery aneurysm; WBC, white blood cell; PLT, platelet count; ALB, albumin; GGT, gamma-glutamyl transferase; HB, hemoglobin Z-score; NR, neutrophil ratio.

Given their clinically plausible temporal relationship, age, symptom count, and pretreatment fever duration were further examined using exploratory SEM to characterize their direct and indirect associations with CAA.

### 3.7 Exploratory SEM and cross-sectional LOESS analysis

**Figure 6:**
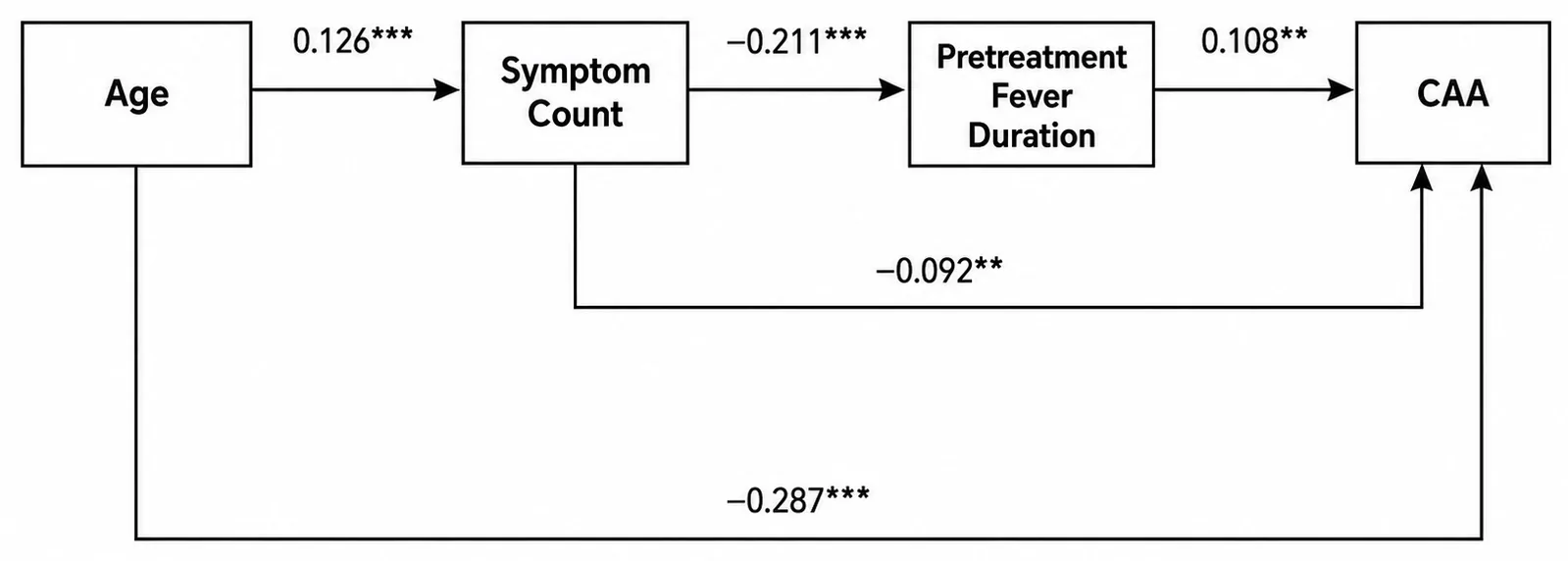
Exploratory structural equation model of associations among age, symptom count, pretreatment fever duration, and CAA. The path diagram shows the prespecified exploratory association structure among age, total symptom count, pretreatment fever duration, and coronary artery aneurysm (CAA). Values adjacent to the arrows are standardized path coefficients (β). Older age was associated with greater symptom count, greater symptom count with shorter pretreatment fever duration, and longer pretreatment fever duration with greater CAA risk. Direct associations of age and symptom count with CAA are also shown. Path directions were specified on clinical grounds, and the model should be interpreted as an exploratory association structure rather than evidence of causal mediation. **P<0.01; ***P<0.001. Abbreviations: CAA, coronary artery aneurysm.

**Figure 7:**
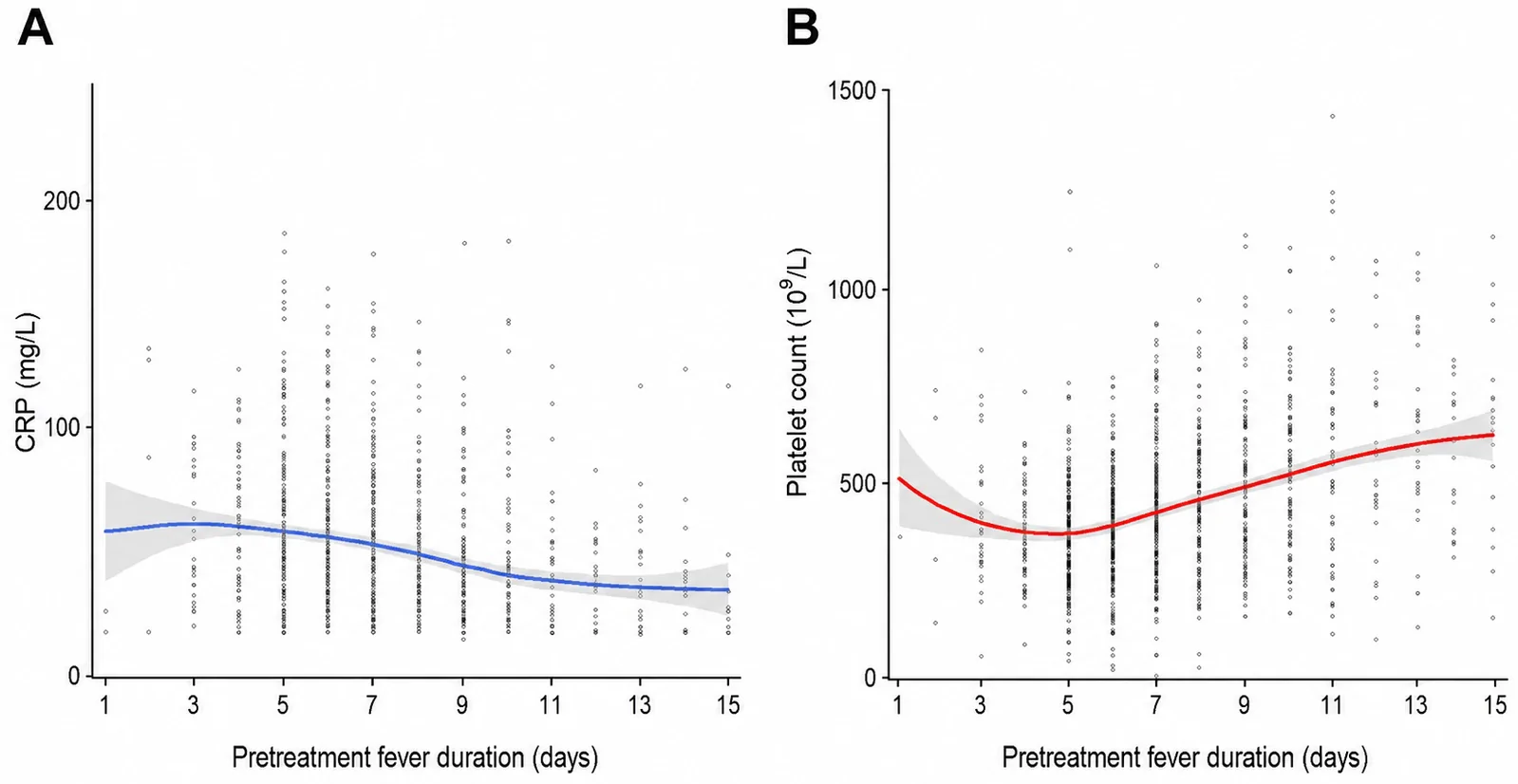
Cross-sectional patterns of C-reactive protein and platelet count according to pretreatment fever duration in iKD. Locally estimated scatterplot smoothing (LOESS) curves illustrate the cross-sectional relationships between pretreatment fever duration and **(A)** C-reactive protein (CRP) and **(B)** platelet count (PLT) among patients with iKD during illness days 1–15. Shaded bands represent 95% confidence intervals, and individual points represent observed patient measurements. The analysis reflects cross-sectional illness-duration-related patterns and should not be interpreted as within-patient longitudinal trajectories or transitions between clusters. Abbreviations: iKD, incomplete Kawasaki disease; CRP, C-reactive protein; PLT, platelet count; IVIG, intravenous immunoglobulin.

Exploratory SEM showed that older age was associated with more clinical manifestations (β=0.126, P<0.001), whereas greater symptom count was associated with shorter pretreatment fever duration (β=−0.211, P<0.001). Age was directly associated with CAA (β=−0.287, P<0.001), while its total indirect association through symptom count and pretreatment fever duration was significant but small (β=−0.015, P = 0.002) (**Table 5**; **Figure 6**). Model fit was χ²=0.712 (df=1, P = 0.399), CFI = 1.000, TLI = 1.010, RMSEA = 0.000, and SRMR = 0.001; given the low degrees of freedom, these indices were interpreted cautiously. LOESS analysis showed an overall decline in CRP with increasing pretreatment fever duration, whereas PLT exhibited a nonlinear pattern, with an early decline followed by a progressive increase after approximately day 5 (**Figure 7**). These patterns were cross-sectional and were not interpreted as longitudinal biomarker trajectories or transitions between clusters.

**Table 5:** Exploratory SEM of associations among age, symptom count, pretreatment fever duration, and coronary artery aneurysm.

Path/effect	B (Unstandardized)	S.E.	p-value	β (Standardized)
Age → Symptom Count	0.078	0.015	<0.001***	0.126
Symptom Count → Pretreatment Fever Duration	-0.713	0.088	<0.001***	-0.211
Age → CAA (Direct)	-0.181	0.016	<0.001***	-0.287
Symptom Count → CAA	-0.094	0.031	0.002**	-0.092
Pretreatment Fever Duration → CAA	0.033	0.010	0.001**	0.108
Indirect association: Age → Symptom Count → CAA	-0.007	0.003	0.009**	-0.012
Indirect association: Age → Symptom Count → Pretreatment Fever Duration → CAA	-0.002	0.001	0.008**	-0.003
Total indirect association (Age → CAA)	-0.009	0.003	0.002**	-0.015
Total association (Age → CAA)	-0.190	0.016	<0.001***	-0.301

This exploratory model evaluates associations among age, symptom count, pretreatment fever duration, and CAA. CAA was modeled as an ordered binary outcome using the WLSMV estimator. Standardized coefficients (β) represent the relative strength of each association. Indirect associations are reported as hypothesis-generating and should not be interpreted as causal mediation. Model fit: scaled χ² = 0.712, df = 1, P = 0.399; CFI = 1.000; TLI = 1.010; RMSEA = 0.000; SRMR = 0.001; N = 1,619. Because the model has only 1 degree of freedom, global fit indices should be interpreted cautiously. *P < 0.05, **P < 0.01, ***P < 0.001. SEM, structural equation modeling; CAA, coronary artery aneurysm; WLSMV, weighted least squares mean- and variance-adjusted estimator.

## 4 Discussion

Our data-driven analysis of 1,619 iKD patients reveals a complex phenotypic landscape defined by four clinically distinct clusters. These findings add to the growing body of evidence supporting the view that KD is not a single disease entity but rather a heterogeneous syndrome. Our clustering structure showed substantial consistency with recently published studies from Western, Chinese, and Japanese populations, while also highlighting patterns particularly relevant to the clinical presentation of iKD (9–12).

C1 represented a predominantly hyper-inflammatory phenotype, characterized by higher CRP, NR, and ESR together with lower ALB and hemoglobin Z-scores. Although C1 did not carry the greatest coronary burden, its inflammatory profile suggests a state of substantial systemic activation. In contrast, C4 occupied the relatively low-inflammatory end of the spectrum, with lower WBC, CRP, and ESR and higher ALB. The coexistence of these inflammatory extremes within iKD emphasizes that an incomplete clinical presentation does not necessarily indicate mild systemic inflammation. Rather, patients meeting iKD criteria may span a broad biological continuum ranging from relatively low inflammatory activity to pronounced systemic inflammatory responses.

C3 was distinguished by prominent hepatobiliary involvement, with markedly elevated ALT and GGT levels, and also showed the highest observed rate of IVIG resistance. This pattern is broadly consistent with previous studies describing hepatic-involved KD subgroups with increased treatment refractoriness (9–12). A large body of evidence has established that liver injury indicators, such as elevated ALT, GGT, and total bilirubin, are independent risk factors for IVIG resistance in KD (27–30). Histopathological studies have demonstrated portal tract edema and inflammatory cell infiltration in KD, while systemic cytokine activation may further contribute to hepatocellular injury (12, 31, 32). Notably, however, the pronounced hepatic involvement and high IVIG-resistance rate in C3 were not accompanied by proportionally severe coronary involvement, suggesting that treatment refractoriness and coronary injury may reflect partially distinct dimensions of KD pathobiology. The relatively short pretreatment fever duration in C3 may have limited cumulative coronary inflammatory exposure despite marked hepatic inflammation, although differences in inflammatory profiles or tissue-specific susceptibility may also contribute. Accordingly, prominent liver involvement may be more informative as a marker of treatment refractoriness than of coronary severity (33, 34).

Among the four phenotypes, C2 was clinically notable for the youngest age, longest pretreatment fever duration, marked thrombocytosis, relatively lower CRP and NR levels, and the greatest post-IVIG coronary burden. C2 had the highest prevalence of CAA, the highest maximum post-IVIG coronary Z-score, and the greatest proportion of medium and giant aneurysms. This pattern is of particular interest because the magnitude of conventional inflammatory markers alone did not parallel coronary severity. Rather than being characterized by the highest CRP or WBC, the highest-risk group combined young age with prolonged pretreatment illness and marked thrombocytosis. These observations suggest that coronary vulnerability in iKD may depend not only on the absolute intensity of systemic inflammation but also on patient age, illness duration, and the evolving hematologic response.

The focused sensitivity analyses provide additional context for the coronary findings and address the potential incorporation bias arising from retrospective iKD classification. After exclusion of the 308 patients whose iKD classification explicitly depended on coronary findings, the major cluster characteristics and between-cluster differences in post-IVIG coronary outcomes were preserved, with C2 retaining the greatest post-IVIG coronary burden. Temporal analysis further showed that pre-IVIG CAA was also most frequent in C2, whereas newly developed CAA after IVIG was uncommon (3.1%) and did not differ significantly across clusters. Among patients with pre-existing CAA, short-term worsening to a higher coronary severity category was similarly uncommon (1.2%), and the median change in maximum coronary Z-score was negative (ΔZ, −0.52 [IQR, −0.80 to 0.05]). This direction of change is consistent with previous longitudinal observations in patients with CAA at diagnosis; Miyata et al. reported a median change in Zmax of −1.3 [−1.8, −0.6] within 2 months among patients treated with IVIG alone, with 52% regressing to Zmax <2 (35). Another cohort of 484 patients with KD reported *de novo* CAA after IVIG in 4.5%, broadly supporting that newly emerging coronary aneurysms after treatment represent a minority of cases (36). However, comparisons of coronary progression require caution. In that study, progressive CAA was defined by progressive dilation across three consecutive echocardiograms with at least an 8% increase in coronary diameter, and follow-up extended well beyond the predischarge period; 33.8% of patients with CAA met that definition (36). This definition is therefore not directly comparable with our conservative short-term definition based on progression to a higher severity category before discharge. Overall, these analyses support the robustness of the primary association between cluster phenotype and post-IVIG coronary burden while also emphasizing that our short observation window cannot characterize later coronary progression.

The association between young age and coronary involvement deserves particular attention. In the multivariable analysis, younger age remained independently associated with post-IVIG CAA even after adjustment for pretreatment fever duration, symptom count, and laboratory covariates. Longer pretreatment fever duration and lower symptom count were also independently associated with CAA. Thus, the analysis does not support a model in which the effect of young age is explained entirely by delayed treatment. Instead, the findings are more compatible with a combination of intrinsic age-related susceptibility and clinical factors related to disease recognition and treatment timing. Previous studies have similarly identified young infants as a particularly vulnerable population for coronary complications (1, 9, 10, 37–40).

Given the clinically plausible temporal relationship among age, clinical completeness, and treatment timing, we further explored these variables using SEM. Older age was associated with a greater number of clinical manifestations, whereas a greater symptom count was associated with shorter pretreatment fever duration. Pretreatment fever duration was in turn positively associated with CAA. The indirect association of age with CAA through symptom count and pretreatment fever duration was statistically significant but small, while a substantially stronger direct association between age and CAA remained. These findings therefore provide only limited support for a pathway linking younger age, fewer clinical manifestations, and longer time to treatment. They are more appropriately interpreted as an exploratory association structure rather than evidence that sparse manifestations causally mediate the coronary risk of young age.

It is also important to distinguish pretreatment fever duration from diagnostic delay. In the present dataset, the interval from first medical contact to KD recognition was unavailable; therefore, longer fever duration before IVIG may reflect several processes, including delayed presentation, sequential emergence of diagnostic features, referral patterns, diagnostic uncertainty, or treatment practice. Our data consequently support an association between longer pretreatment fever duration and coronary risk but do not establish physician-related or patient-related diagnostic delay. This distinction is particularly important when interpreting the sparse clinical presentations observed in younger infants.

The LOESS analysis revealed distinct cross-sectional patterns of CRP and PLT across increasing fever duration. CRP showed an overall downward pattern across increasing pretreatment fever duration, whereas PLT displayed a nonlinear pattern with an early nadir followed by a progressive increase later in the illness course, a pattern broadly consistent with the recognized evolution of inflammatory and hematologic responses across the acute and subacute phases of KD (1, 37, 41). These findings raise the possibility that some of the contrasting CRP–PLT profiles observed across clusters may partly reflect illness-duration-related variation rather than completely discrete biological states. However, because the LOESS analysis pooled cross-sectional observations rather than paired longitudinal measurements within individual patients, it cannot establish a sequential transition between clusters. The observed pattern should therefore be considered hypothesis-generating and requires confirmation using longitudinal biomarker measurements.

The whole-KD analysis provided an important context for interpreting the iKD clusters. Application of the same pretreatment-only clustering framework to the entire KD cohort independently identified four clusters. Although the whole-KD clusters did not show direct one-to-one correspondence with the iKD cluster solution, iKD was unevenly distributed across these clusters and was most strongly represented in one cluster. These findings suggest that incomplete presentations are non-randomly distributed within the broader phenotypic heterogeneity of KD.

Our subphenotyping results therefore corroborate previous data-driven studies that have identified recurrent young-age, inflammatory, hepatobiliary, and relatively mild phenotypes across diverse KD populations (9–12). The consistency across studies supports the reproducibility of several major axes of KD heterogeneity, while the differences in cluster composition emphasize that cluster labels should not be interpreted as fixed disease entities. Rather, they may represent recurring combinations of age, systemic inflammation, organ involvement, and illness duration whose relative prominence varies across populations and study designs.

These findings may have implications for future risk assessment in iKD. In particular, young infants with prolonged pretreatment fever and marked thrombocytosis may warrant heightened attention even when CRP is not exceptionally elevated, because a high-PLT/moderate-CRP pattern may coexist with substantial post-IVIG coronary involvement. Similarly, prominent hepatobiliary involvement may identify patients at greater risk of IVIG resistance even when coronary involvement is not disproportionately severe. However, the present study was not designed as a prospective prediction study, and cluster membership should not be interpreted as a validated bedside risk classifier. Whether these phenotypic patterns can prospectively improve treatment selection or identify patients who benefit from intensified therapy requires prospective validation.

The major strengths of this study include the large, multicenter iKD cohort and the application of advanced statistical techniques, SEM and LOESS regression, to characterize association structures and illness-duration-related biomarker patterns.Several limitations nevertheless warrant consideration. Most importantly, iKD classification was retrospectively reassessed using documented clinical manifestations and the 2020 Japanese diagnostic criteria, raising potential concerns regarding misclassification and incorporation or selection bias when coronary findings contributed to retrospective diagnosis. The focused sensitivity analysis excluded the 308 patients whose iKD classification explicitly depended on coronary findings; preservation of the major cluster characteristics and post-IVIG coronary outcome differences in the remaining 1,311 patients reduces, although cannot completely eliminate, this concern. Residual classification uncertainty is greatest for highly incomplete presentations with ≤2 documented principal features, for whom the Japanese guideline does not specify a fixed supportive-feature threshold. Moreover, because manifestations may emerge sequentially and historical records were not prospectively standardized for research, transient or subsequently emerging features may have been incompletely documented, potentially underestimating symptom counts in some patients. In addition, echocardiographic timing and frequency were not prospectively standardized across centers. Although the sensitivity analysis separated pre-IVIG abnormalities from newly developed and worsening abnormalities after IVIG, follow-up for the present outcome analysis extended only to hospital discharge and may therefore have missed subsequent coronary progression during the subacute phase. Worsening was also defined conservatively as progression to a higher severity category, although ΔZ was additionally examined to capture continuous changes in coronary dimensions. Pretreatment fever duration should likewise be interpreted as a surrogate for the interval before treatment rather than a direct measure of diagnostic delay, because the timing of first medical contact and definitive diagnosis was unavailable. Finally, the cross-sectional nature of the LOESS analysis precludes inference of within-patient biomarker trajectories, and the retrospective observational design precludes causal interpretation of the SEM pathways.

In summary, this multicenter study identified four clinically distinct subphenotypes within iKD. The young-age/thrombocytosis phenotype showed the greatest coronary burden, which remained evident after exclusion of coronary-dependent iKD cases, whereas the hepatobiliary-involved phenotype showed the highest IVIG-resistance rate, highlighting substantial clinical heterogeneity within iKD and the need for prospective validation of these phenotypes.

## 5 Conclusions

A young-age/thrombocytosis phenotype was associated with the greatest post-IVIG coronary burden, whereas a hepatobiliary-involved phenotype showed the highest observed IVIG-resistance rate without proportionately severe coronary involvement. Sensitivity analyses showed that the major phenotypic patterns were preserved after exclusion of coronary-dependent iKD cases. Younger age, longer pretreatment fever duration, fewer clinical manifestations, lower ALB, lower hemoglobin Z-score, and lower NR were independently associated with post-IVIG CAA. Exploratory SEM suggested only a small indirect association linking age, symptom count, and pretreatment fever duration, supporting a multifactorial rather than exclusively delay-mediated explanation for the coronary vulnerability of young patients. The whole-KD analysis further showed that iKD was unevenly distributed across the broader phenotypic heterogeneity of KD. Prospective longitudinal studies are required to validate these phenotypes and determine their clinical utility.

## Data Availability

The data can be obtained with reasonable reasons from corresponding authors. Requests to access the datasets should be directed to Fengchuan Jing, 987348411@qq.com.
