# Supplementary material for: Decoding clinical heterogeneity and age-related coronary risk in incomplete Kawasaki disease: a multicenter cluster analysis and structural equation modeling study
Source: Front Immunol. 2026 Sep 18;17:1908370. doi: 10.3389/fimmu.2026.1908370 (PMC13592195; doi:10.3389/fimmu.2026.1908370)
Supplement: Supplementary file 1 [file Supplementaryfile1.docx]

Supplementary Material

# 1 Supplementary Data

**1 Supplementary Text S1: Detailed Study Protocols**

**1.1 Eligibility Criteria**

Patients were enrolled if they met the following clinical prerequisites: (1) a confirmed diagnosis of incomplete Kawasaki disease (iKD); (2) receipt of standard primary treatment [2 g/kg intravenous immunoglobulin (IVIG) and aspirin]; and (3) availability of echocardiographic data sufficient for post-IVIG coronary outcome assessment.

Subjects were excluded if they presented with pre-existing cardiovascular conditions, inherited metabolic syndromes, or concurrent malignancies. Furthermore, those who had been treated with immunomodulatory agents (e.g., corticosteroids or biologics) within 3 months prior to the acute phase were omitted from the analysis.

**1.2 Coronary Artery Severity Stratification**

Based on the maximum post-IVIG Z-score recorded after completion of the initial IVIG infusion and before hospital discharge, coronary status was categorized into five severity tiers.

Normal Coronary: Zmax < 2.0;

Coronary Dilation: 2.0 ≤ Zmax < 2.5;

Small CAA: 2.5 ≤ Zmax < 5.0;

Medium CAA: 5.0 ≤ Zmax < 10.0 (and internal diameter < 8 mm);

Giant CAA: Zmax ≥ 10.0 (or internal diameter ≥ 8 mm).

Post-IVIG CAA was defined as a maximum coronary Z-score ≥2.5 during this predefined assessment window.

**2 Supplementary Tables**

**Supplementary Table S1. Clinical Characteristics, Laboratory Profiles, Treatment Response, and Coronary Outcomes Across the Four KD Clusters**

| Variable | Overall (n=6264) | 1 (n=3287) | 2 (n=1541) | 3 (n=851) | 4 (n=585) | P-value |
| --- | --- | --- | --- | --- | --- | --- |
| Age (Years, median [IQR]) | 1.4 [0.8,2.1] | 1.2 [0.6,1.8] | 1.3 [0.8,1.9] | 1.3 [0.9,1.9] | 4.9 [4.0,6.1] | <0.001*** |
| Fever Duration (Days, median [IQR]) | 6 [5,8] | 6 [5,7] | 7 [6,10] | 5 [5,6] | 6 [5,7] | <0.001*** |
| WBC (10^9/L, median [IQR]) | 14.1 [10.8,18.1] | 15.9 [12.5,20.1] | 11.7 [8.9,14.7] | 13.8 [11.0,17.6] | 13.3 [9.5,15.7] | <0.001*** |
| PLT (10^9/L, median [IQR]) | 401.0 [310.0,510.0] | 411.0 [314.0,509.2] | 438.0 [330.0,578.0] | 359.5 [285.5,433.2] | 352.0 [280.0,428.0] | <0.001*** |
| CRP (mg/L, median [IQR]) | 43.0 [23.0,67.0] | 54.0 [36.0,79.0] | 17.0 [8.0,30.0] | 51.0 [33.0,71.2] | 45.0 [24.8,67.2] | <0.001*** |
| ALB (g/L, median [IQR]) | 37.7 [34.1,40.8] | 35.8 [32.1,39.2] | 40.8 [38.0,43.1] | 37.7 [34.9,40.2] | 38.4 [35.2,41.4] | <0.001*** |
| ALT (U/L, median [IQR]) | 29.8 [17.4,60.9] | 29.2 [17.7,49.0] | 21.8 [14.2,32.0] | 137.8 [85.0,236.6] | 28.1 [15.0,54.9] | <0.001*** |
| GGT (U/L, median [IQR]) | 33.0 [14.9,94.2] | 37.0 [16.4,82.4] | 17.0 [11.0,31.1] | 186.2 [124.0,282.0] | 19.0 [11.9,64.5] | <0.001*** |
| Post-IVIG maximum coronary Z-score (median [IQR]) | 2.48 [1.78,3.34] | 2.63 [1.88,3.47] | 2.39 [1.78,3.33] | 2.38 [1.74,3.21] | 1.98 [1.38,2.55] | <0.001*** |
| Hemoglobin Z-score (median [IQR]) | -2.25 [-3.33,-1.33] | -2.75 [-3.67,-1.87] | -1.62 [-2.50,-0.75] | -1.47 [-2.27,-0.67] | -2.78 [-3.88,-1.86] | <0.001*** |
| NR (%, median [IQR]) | 64.0 [54.0,74.0] | 66.0 [58.0,75.0] | 51.0 [41.0,61.0] | 71.0 [63.0,79.0] | 76.0 [70.0,84.0] | <0.001*** |
| ESR (mm/h, median [IQR]) | 64.0 [44.0,85.0] | 66.0 [45.0,89.0] | 53.0 [33.0,72.0] | 68.5 [51.0,86.0] | 73.0 [59.2,91.0] | <0.001*** |
| CAA n (%) | 3085 (49.2) | 1812 (55.1) | 726 (47.1) | 399 (46.9) | 148 (25.3) | <0.001*** |
| Detailed severity n (%) |  |  |  |  |  | <0.001†*** |
| Normal Coronary | 2058 (32.9) | 930 (28.3) | 525 (34.1) | 304 (35.7) | 299 (51.1) |  |
| Coronary Dilation | 1121 (17.9) | 545 (16.6) | 290 (18.8) | 148 (17.4) | 138 (23.6) |  |
| Small CAA | 2655 (42.4) | 1542 (46.9) | 603 (39.1) | 374 (43.9) | 136 (23.2) |  |
| Medium CAA | 380 (6.1) | 250 (7.6) | 95 (6.2) | 25 (2.9) | 10 (1.7) |  |
| Giant CAA | 50 (0.8) | 20 (0.6) | 28 (1.8) | 0 (0.0) | 2 (0.3) |  |
| Resistance n (%) | 466 (7.4) | 260 (7.9) | 69 (4.5) | 95 (11.2) | 42 (7.2) | <0.001*** |
| Incomplete KD n (%) | 1619 (25.8) | 769 (23.4) | 559 (36.3) | 170 (20.0) | 121 (20.7) | <0.001*** |
| Symptom count (median [IQR]) | 3 [2,4] | 3 [2,4] | 2 [1,3] | 3 [2,4] | 3 [2,4] | <0.001*** |

Notes: Continuous variables with non-normal distributions are presented as median [interquartile range, IQR], and differences among groups were evaluated using the Kruskal-Wallis H test. Categorical variables are presented as frequencies (%) and were compared using the Chi-squared test or Fisher’s exact test. * P < 0.05, ** P < 0.01, *** P < 0.001. † P-value was calculated for the overall distribution of detailed coronary severity grades across the four clusters. The clustering model included Age, fever duration, WBC, PLT, CRP, ALB, ALT, GGT, hemoglobin Z-score, NR, and ESR; coronary Z-score and subsequent outcomes were excluded from cluster derivation. Abbreviations: KD, Kawasaki disease; iKD, incomplete Kawasaki disease; IQR, interquartile range; WBC, white blood cell; PLT, platelet; CRP, C-reactive protein; ALB, albumin; ALT, alanine aminotransferase; GGT, gamma-glutamyl transferase; CAA, coronary artery aneurysm; NR, neutrophil ratio; ESR, erythrocyte sedimentation rate; IVIG, intravenous immunoglobulin.

**Supplementary Table S2. Clinical Characteristics, Laboratory Profiles, Treatment Response, and Coronary Outcomes Across the Four iKD Clusters in the Coronary-Independent Sensitivity Cohort**

| Variable | Overall (n=1311) | 1 (n=474) | 2 (n=418) | 3 (n=116) | 4 (n=303) | P-value |
| --- | --- | --- | --- | --- | --- | --- |
| Age (Years, median [IQR]) | 1.3 [0.6, 2] | 1.3 [0.6, 2.7] | 1.05 [0.6, 1.6] | 1.35 [0.9, 1.92] | 1.6 [1, 2.3] | <0.001*** |
| Fever Duration (Days, median [IQR]) | 7 [6, 9] | 6 [5, 7] | 9 [7, 11] | 5 [5, 6] | 8 [6, 10] | <0.001*** |
| WBC (10^9/L, median [IQR]) | 13.51 [9.88, 17.73] | 15.39 [11.16, 19.73] | 15.02 [12.3, 18.79] | 13.81 [11.09, 16.35] | 9.26 [6.88, 11.93] | <0.001*** |
| PLT (10^9/L, median [IQR]) | 418 [312, 544.5] | 361 [264, 450.2] | 586.5 [459.2, 720.8] | 347 [283.5, 414.5] | 382.5 [282.5, 487.2] | <0.001*** |
| CRP (mg/L, median [IQR]) | 35 [17, 59] | 56 [35, 84] | 35 [20, 50] | 51 [36, 74] | 11 [8, 20] | <0.001*** |
| ALB (g/L, median [IQR]) | 38.5 [35.1, 41.9] | 37.8 [34, 41.1] | 37.2 [34.5, 40] | 37.6 [34.5, 41.2] | 41.7 [39.4, 44.2] | <0.001*** |
| ALT (U/L, median [IQR]) | 26 [16.4, 46.4] | 28.3 [18.5, 44.3] | 23.9 [14.4, 36] | 171.1 [106.3, 269.2] | 21.3 [14, 30.9] | <0.001*** |
| GGT (U/L, median [IQR]) | 25 [13, 65] | 28.7 [14.1, 62.2] | 31.1 [14.6, 62] | 202 [125, 294.5] | 13 [10, 19.1] | <0.001*** |
| Maximum post-IVIG Z-score (median [IQR]) | 2.08 [1.52, 2.86] | 1.96 [1.51, 2.63] | 2.35 [1.63, 3.61] | 1.97 [1.43, 2.64] | 1.97 [1.46, 2.6] | <0.001*** |
| Hemoglobin Z-score (median [IQR]) | -2.12 [-3.12, -1.12] | -2.25 [-3.25, -1.25] | -2.75 [-3.75, -2] | -1.88 [-2.56, -0.69] | -1.38 [-2.12, -0.38] | <0.001*** |
| NR (%, median [IQR]) | 60.99 [49.98, 70.97] | 69.01 [60.76, 77] | 57.03 [49, 66.01] | 70.01 [61, 78.47] | 48.05 [37.06, 58] | <0.001*** |
| ESR (mm/h, median [IQR]) | 63 [42.5, 86] | 61 [46, 78] | 85 [64, 105.2] | 69 [47, 91] | 40 [23, 60] | <0.001*** |
| CAA n (%) | 591 (45.1) | 182 (38.4) | 241 (57.7) | 48 (41.4) | 120 (39.6) | <0.001*** |
| Detailed severity n (%) |  |  |  |  |  | <0.001†*** |
| Normal Coronary | 530 (40.4) | 218 (46.0) | 127 (30.4) | 45 (38.8) | 140 (46.2) |  |
| Coronary Dilation | 190 (14.5) | 74 (15.6) | 50 (12.0) | 23 (19.8) | 43 (14.2) |  |
| Small CAA | 504 (38.4) | 166 (35.0) | 184 (44.0) | 44 (37.9) | 110 (36.3) |  |
| Medium CAA | 81 (6.2) | 15 (3.2) | 52 (12.4) | 4 (3.4) | 10 (3.3) |  |
| Giant CAA | 6 (0.5) | 1 (0.2) | 5 (1.2) | 0 (0.0) | 0 (0.0) |  |
| Resistance n (%) | 111 (8.5) | 60 (12.7) | 21 (5.0) | 17 (14.7) | 13 (4.3) | <0.001*** |
| Male Gender n (%) | 832 (63.5) | 319 (67.3) | 262 (62.7) | 62 (53.4) | 189 (62.4) | 0.040* |
| Symptom count (median [IQR]) | 1 [1, 2] | 1 [1, 2] | 1 [1, 2] | 1 [1, 2] | 1 [0, 2] | <0.001*** |
| Oral mucosal changes n (%) | 333 (25.4) | 130 (27.4) | 95 (22.7) | 33 (28.4) | 75 (24.8) | 0.356 |
| Conjunctival injection n (%) | 640 (48.8) | 242 (51.1) | 207 (49.5) | 63 (54.3) | 128 (42.2) | 0.053 |
| Rash n (%) | 383 (29.2) | 155 (32.7) | 91 (21.8) | 57 (49.1) | 80 (26.4) | <0.001*** |
| Acral changes n (%) | 338 (25.8) | 135 (28.5) | 104 (24.9) | 23 (19.8) | 76 (25.1) | 0.240 |
| Cervical lymphadenopathy n (%) | 111 (8.5) | 51 (10.8) | 25 (6.0) | 10 (8.6) | 25 (8.3) | 0.087 |

Notes: Continuous variables with non-normal distributions are presented as median [interquartile range, IQR], and differences among groups were evaluated using the Kruskal-Wallis H test. Categorical variables are presented as frequencies (%) and were compared using the Chi-squared test or Fisher’s exact test. * P < 0.05, ** P < 0.01, *** P < 0.001. † P-value was calculated for the overall distribution of detailed coronary severity grades across the four clusters. Abbreviations: iKD, incomplete Kawasaki disease; IQR, interquartile range; WBC, white blood cell; PLT, platelet; CRP, C-reactive protein; ALB, albumin; ALT, alanine aminotransferase; GGT, gamma-glutamyl transferase; CAA, coronary artery aneurysm; NR, neutrophil ratio; ESR, erythrocyte sedimentation rate.

**Supplementary Table S3. Temporal Coronary Outcomes Across the Four iKD Clusters in the Coronary-Independent Sensitivity Cohort**

| Variable | Overall (n=1311) | 1 (n=474) | 2 (n=418) | 3 (n=116) | 4 (n=303) | P-value |
| --- | --- | --- | --- | --- | --- | --- |
| Pre-IVIG CAA, n/N (%) | 568/1311 (43.3) | 171/474 (36.1) | 235/418 (56.2) | 46/116 (39.7) | 116/303 (38.3) | <0.001*** |
| Newly developed CAA among pre-IVIG non-CAA, n/N (%) | 23/743 (3.1) | 11/303 (3.6) | 6/183 (3.3) | 2/70 (2.9) | 4/187 (2.1) | 0.827 |
| Worsening to a higher severity category among pre-IVIG CAA, n/N (%) | 7/568 (1.2) | 1/180 (0.6) | 4/216 (1.9) | 0/44 (0) | 2/128 (1.6) | 0.567 |
| Change in maximum coronary Z-score (ΔZ), median [IQR]† | -0.52 [-0.80, 0.05] | -0.53 [-0.75, 0.04] | -0.50 [-0.90, 0.05] | -0.55 [-0.89, -0.20] | -0.48 [-0.71, 0.05] | 0.497 |

Notes: Categorical variables are presented as n/N (%) and were compared across clusters using the Chi-squared test or Fisher’s exact test, as appropriate. Newly developed CAA was defined as a post-IVIG coronary Z-score ≥2.5 among patients with a pre-IVIG Z-score <2.5. Worsening of pre-existing CAA was conservatively defined as progression to a higher predefined coronary severity category during the post-IVIG predischarge period. † ΔZ was calculated as the maximum post-IVIG coronary Z-score minus the maximum pre-IVIG coronary Z-score among patients with pre-IVIG CAA; values are presented as median [IQR] and were compared across clusters using the Kruskal-Wallis H test. * P < 0.05, ** P < 0.01, *** P < 0.001. Abbreviations: iKD, incomplete Kawasaki disease; IVIG, intravenous immunoglobulin; CAA, coronary artery aneurysm; IQR, interquartile range.

**3 Supplementary Figures**

**Supplementary Figure S1. Determination of the Optimal Number of Clusters in the Whole KD Cohort.** Elbow plot showing the total within-cluster sum of squares across candidate cluster numbers in the entire Kawasaki disease (KD) cohort (n=6,264). The red dashed line indicates the selected four-cluster solution (k=4). Abbreviation: KD, Kawasaki disease.

**Supplementary Figure S2. Principal Component and Heatmap Visualization of Clinical Heterogeneity in the Whole KD Cohort.** (A) Standardized heatmap showing the clinical and laboratory profiles of the four independently derived whole-KD clusters. Cell values represent standardized cluster-level values, with red indicating relatively higher and blue indicating relatively lower levels. (B) PCA biplot showing the distribution of the four whole-KD clusters, with arrows representing the loadings of the 11 pretreatment variables. The whole-KD clusters were derived independently and were not assumed to correspond directly to the four iKD clusters.

**Supplementary Figure S3. Distribution of iKD and Coronary Outcomes Across the Four Whole-KD Clusters.** Stacked bar charts show (A) the proportions of incomplete KD (iKD) and complete KD (cKD), (B) the proportions of patients with and without coronary artery aneurysm (CAA), and (C) the distribution of coronary severity categories across the four independently derived whole-KD clusters. iKD accounted for 23.4%, 36.3%, 20.0%, and 20.7% of Clusters 1–4, respectively. Coronary status was evaluated using post-IVIG coronary measurements. Abbreviations: KD, Kawasaki disease; iKD, incomplete Kawasaki disease; cKD, complete Kawasaki disease; CAA, coronary artery aneurysm; IVIG, intravenous immunoglobulin.

**Supplementary Figure S4.** **Standardized Heatmap of Clinical and Laboratory Profiles Across the Four iKD Clusters in the Restricted Sensitivity Cohort.** The heatmap shows the standardized cluster-level mean values of the 11 pretreatment clinical and laboratory variables used for clustering after exclusion of patients whose retrospective iKD classification explicitly depended on coronary findings. Red indicates relatively higher standardized values and blue indicates relatively lower values. Hierarchical dendrograms illustrate similarities among variables and cluster-level profiles. Abbreviations: iKD, incomplete Kawasaki disease; WBC, white blood cell count; PLT, platelet count; CRP, C-reactive protein; ALB, albumin; ALT, alanine aminotransferase; GGT, gamma-glutamyl transferase; HB, hemoglobin Z-score; NR, neutrophil ratio; ESR, erythrocyte sedimentation rate; Day, pretreatment fever duration.
